# mTORC1 activity is essential for erythropoiesis and B cell lineage commitment

**DOI:** 10.1038/s41598-019-53141-1

**Published:** 2019-11-15

**Authors:** Natasha Malik, Karen M. Dunn, Jennifer Cassels, Jodie Hay, Christopher Estell, Owen J. Sansom, Alison M. Michie

**Affiliations:** 1 Institute of Cancer Sciences, College of Medicine, Veterinary and Life Sciences, University of Glasgow, Glasgow, UK; 2 Cancer Research UK Beatson Institute, Garscube Estate, Glasgow, UK

**Keywords:** Haematopoietic stem cells, Lymphopoiesis

## Abstract

Mechanistic target of rapamycin (mTOR) is a serine/threonine protein kinase that mediates phosphoinositide-3-kinase (PI3K)/AKT signalling. This pathway is involved in a plethora of cellular functions including protein and lipid synthesis, cell migration, cell proliferation and apoptosis. In this study, we proposed to delineate the role of mTORC1 in haemopoietic lineage commitment using knock out (KO) mouse and cell line models. *Mx1*-cre and *Vav*-cre expression systems were used to specifically target *Raptor*^*fl/fl*^ (mTORC1), either in all tissues upon poly(I:C) inoculation, or specifically in haemopoietic stem cells, respectively. Assessment of the role of mTORC1 during the early stages of development in *Vav*-cre^+^*Raptor*^*fl/fl*^ mice, revealed that these mice do not survive post birth due to aberrations in erythropoiesis resulting from an arrest in development at the megakaryocyte-erythrocyte progenitor stage. Furthermore, *Raptor*-deficient mice exhibited a block in B cell lineage commitment. The essential role of Raptor (mTORC1) in erythrocyte and B lineage commitment was confirmed in adult *Mx1-cre*^+^*Raptor*^*fl/fl*^ mice upon cre-recombinase induction. These studies were supported by results showing that the expression of key lineage commitment regulators, *GATA1*, *GATA2* and *PAX5* were dysregulated in the absence of mTORC1-mediated signals. The regulatory role of mTOR during erythropoiesis was confirmed *in vitro* by demonstrating a reduction of K562 cell differentiation towards RBCs in the presence of established mTOR inhibitors. While mTORC1 plays a fundamental role in promoting RBC development, we showed that mTORC2 has an opposing role, as *Rictor-*deficient progenitor cells exhibited an elevation in RBC colony formation *ex vivo*. Collectively, our data demonstrate a critical role played by mTORC1 in regulating the haemopoietic cell lineage commitment.

## Introduction

Recent studies of erythroid differentiation have revealed differences in the lineage potential of distinct cellular subsets between fetal and adult progenitor populations, with a higher proportion of megakaryocyte-erythroid progenitors arising from multipotent and oligopotent progenitor subsets during fetal haemopoiesis, while adult erythroid-committed cells are derived predominantly from unipotent progenitors^1^. These studies suggest that the erythroid cell lineage may be differentially regulated during ontogeny. Multipotent progenitor (MPP) cells give rise to lymphoid-primed multipotent progenitors (LMPPs) and oligopotent common myeloid progenitors (CMPs). LMPPs have the capacity to differentiate towards common lymphoid progenitors (CLPs) mainly, with reduced megakaryocyte-erythroid progenitor (MEP) potential, while CMPs branch towards MEPs and granulocyte-macrophage progenitor (GMP) cells^2^. MEPs further commit to the erythroid lineage through formation of burst-forming unit-erythroid (BFU-E) and then colony-forming unit-erythroid (CFU-E) cells, which then progress through erythroblast stages, reticulocyte maturation and terminal differentiation into red blood cells (RBCs)^3^.

Erythropoiesis is governed by the complex regulation of key transcription factors (TFs), enabling a balance between self-renewal and differentiation of erythrocyte progenitors and terminal erythroblasts. The zinc-finger TFs *GATA1* and *GATA2* play critical and non-redundant roles during erythroid maturation. *GATA2* is expressed in HSCs and early progenitor populations regulating the expression of self-renewal genes, and genes responsible for initiating *GATA1* expression. *GATA1* plays a vital role in erythroid differentiation, sustaining its own expression and suppressing *GATA2* expression, a process called *GATA* factor switching. *GATA1*-deficient (*GATA1*^−/−^) murine embryos give rise to lymphoid cells and non-haemopoietic tissues, but not a mature erythroid population, resulting from a block at the pro-erythroblast stage due to increased apoptosis^4^. *Ikaros* also plays a role in fetal and adult erythropoiesis and erythroid lineage commitment, with *Ikaros* gene silencing leading to an irreversible switch to the myeloid lineage^5^. *PU.1* TF is an established master regulator in haemopoiesis involved in primitive cell fate decisions. *PU.1* expression levels determine myeloid and lymphoid cell fates: higher expression of *PU.1* leads to myeloid cell fate while a lower expression to a lymphoid fate^6^. The myeloid cell fate is not solely regulated by *PU.1* expression, but also the inhibition of *GATA1*^7^ and the expression of *CEBPα*^8^. *PU.1* and *GATA1* physically interact to regulate lineage fate where upregulation of *GATA1* inhibits *PU.1* transcription and promotes erythroid lineage differentiation^9^, while expression of *PU.1* inhibits *GATA1* expression promoting myeloid lineage fate^10^. *GATA1* binds to the promoter region of erythroid-Krüppel-like factor (*e-KLF/Klf1*)^11^ which is vital for erythropoiesis, regulating MEP lineage fate decisions during erythrocyte development and globin switching from *γ-* to *β-GLOBIN* during erythrocyte maturation^12^. In humans, *β-GLOBIN* is expressed when erythropoiesis moves to the bone marrow (BM). Initially, the yolk-sac expresses *ε-GLOBIN*, followed by the expression of *γ-GLOBIN* in the fetal liver (FL) and spleen. Therefore, *e-KLF* mediated *β-GLOBIN* expression is vital for mature erythrocyte development^13^. While *e-KLF* plays a role in erythropoiesis, *KLF2* is involved in endothelial growth, vascular remodeling and inflammation responses^14^, which is vital for embryonic development. Lower expression levels of *PU.1* skew progenitors towards a lymphoid lineage enhancing *E2A* expression, a TF involved at the earliest stages of B cell development. Lack of *E2A* leads to a block in B cell development at the pre-proB and pro-B stages^15^. *E2A* drives the expression of early B cell factor 1 (*EBF1*), which together with *E2A*, regulate *PAX5* and *RAG* genes responsible for B cell lineage commitment and V(D)J recombination to form the pre-B cell receptor complex on pre-B cells^16^.

The mTOR/AKT signaling pathway has been shown to play a vital role in haemopoietic lineage development and maturation. The mTOR pathway is activated by a variety of growth factor receptors and nutrients including glucose, iron and amino acids. mTOR forms two different complexes – mTORC1 and mTORC2. mTORC1 is composed of 6 proteins and mTORC2 of 7 proteins. Of these, mTOR kinase is common, along with GβL, DEPTOR, and the TTI1/TEL2 complex. The subunits that make the respective complexes unique are RAPTOR (rapamycin TOR-sensitive) and PRAS40 for mTORC1 and RICTOR (rapamycin TOR-insensitive), mSIN1, and PROTOR1/2 for mTORC2. AKT lies upstream of mTORC1 and downstream of mTORC2 thus playing a crucial role in mTOR pathway regulation^17^. Downstream of mTORC1, S6 kinase 1 (S6K1) inhibits mTORC2 activity, thereby creating a negative feedback loop and another regulatory mechanism for this pathway^18^.

A critical role of mTORC1 has been identified in erythropoiesis whereby mTORC1 is regulated by dietary iron and *Raptor* ablation at the haemopoietic stem cell (HSC) stage leads to perinatal lethality^19^. Furthermore *Raptor* KO and overexpression leads to microcytic and macrocytic anaemia respectively^19^. However, discrepancies in the field remain, as there is research demonstrating that mTORC1 inhibition does not cause anaemia^20^ and that mTORC1 inhibition improves anaemia in a sickle cell disease model^21^. In this study, we address the role of *Raptor* (mTORC1) in normal haemopoietic lineage commitment both in fetal and adult developmental stages, using the cre-LoxP system to excise *Raptor* (mTORC1) in adult mice (*Mx1-*cre) and at the HSC stage (*Vav-*cre) in embryos, as well as the K562 cell line, to address the role of mTOR-mediated signals during RBC differentiation.

## Results

### mTORC1 plays a critical role in B cell and RBC lineage development *in vivo*

**Figure 1:**
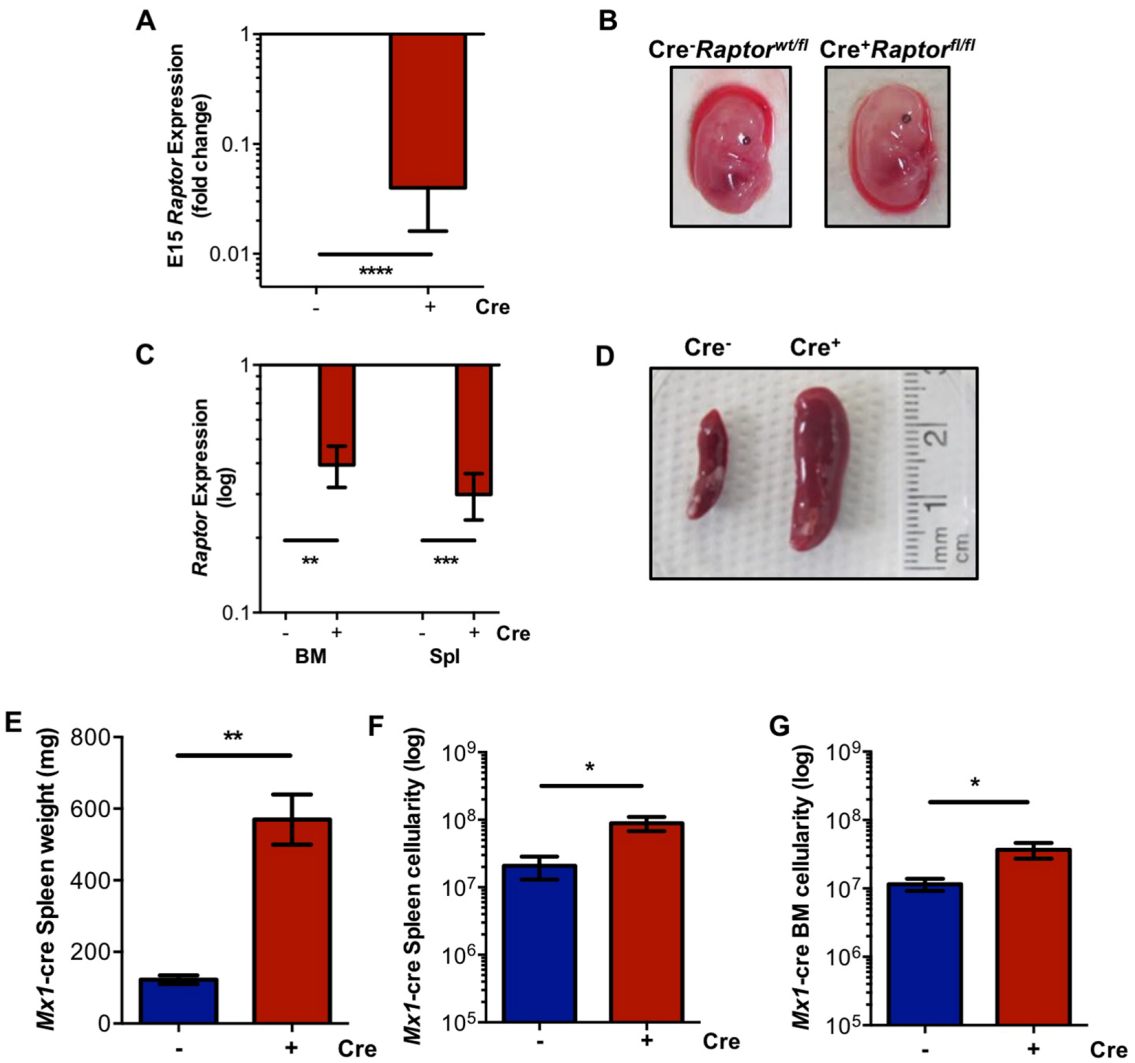
*Vav*-cre^+^*Raptor*^*fl/fl*^ mice die perinatally in the absence of *raptor* expression. (**A**) Gene expression data showing expression of *Raptor* with cre expression in *Vav-cre*^+^*Raptor*^*fl/fl*^ (*Vav-Raptor* KO) compared to *Vav*-cre^−^*Raptor*^*fl/fl*^ (control) E15 FL (n = 6). (**B**) Picture showing the difference in pallor in E13 FL between *Vav*-cre^−^*Raptor*^wt/fl^ and *Vav-*KO fetal mice; **C**. Gene expression of *Raptor* in the BM and spleen in *Mx1*-cre^−^ and *Mx1-*cre^+^*Raptor*^*fl/fl*^ mice (red, *Mx1-Raptor* cKO; n = 5); **D**. Picture showing splenomegaly in *Mx1*-*Raptor* cKO mice compared to *Mx1*-*Raptor* control; Graphs showing spleen weight (mg) **(E)**, spleen cellularity **(F)** and BM cellularity (**G**) of *Mx1*-*Raptor* control (blue) and *Mx1-Raptor* cKO mice (n ≥ 4). All *Mx1*-cre mice were given 4 doses of poly(I:C) and assessed 5 wk post inoculation. Data are expressed as mean ± SEM, t-test (unpaired) (p * ≤ 0.05; ** ≤ 0.001; *** ≤ 0.0001; **** ≤ 0.00001.

Genotyping analyses of *Vav*-cre^+/−^ x *Raptor*^*wt/fl*^ breedings revealed that the mice exhibiting the *Vav*-*Raptor* KO (*Vav*-cre^−/+^*Raptor*^*fl/fl*^) did not reach weaning age (4 wk), while being present at normal ratios during gestation, suggesting that *Vav*-cre^−/+^*Raptor*^*fl/fl*^ mice died perinatally (Table 1). Analysis of embryos (E13–17) revealed a significant downregulation of *Raptor* expression in FL isolated from *Vav*-*Raptor* KO mice as expected, and the embryos were pale during gestation compared to *Vav*-cre^−/−^*Raptor*^*fl/fl*^ (*Vav-Raptor* control) (Fig. 1A,B, & data not shown). Induction of *Raptor* excision with poly(I:C) in adult *Mx1*-*Raptor* cKO mice revealed a significant downregulation in *Raptor* expression in the BM and spleen (Fig. 1C) together with an increase in splenic weight and cellularity, and BM cellularity in *Mx1-*cre^+^*Raptor*^*fl/fl*^ mice (*Mx1-Raptor* cKO) compared to *Mx1-*cre^−^*Raptor*^*fl/fl*^ mice (*Mx1*-*Raptor* control) (Fig. 1D–G).

**Table 1:** Deletion of *Raptor* in the haemopoietic lineages leads to death prior to weaning.

Age	Medelian Ratio	Cre^−^*Raptor*^wt/fl^	Cre^−^ *Raptor*^fl/fl^	Cre^+^ *Raptor*^wt/fl^	Cre^+^ *Raptor*^fl/fl^	Chi^2^ Value	*p* Value
Weaning	Expected	6.5	6.5	6.5	6.5	14.92	**
	Actual	13	9	4	0		
E13	Expected	12.25	12.25	12.25	12.25	2.92	0.50
	Actual	12	8	15	14		

The expected mendelian ratios and actual genotyped ratios of *Vav*-cre^+/−^ *Raptor*^*wt/fl*^ mouse matings are shown at weaning (4 wk; n = 26) and at embryonic day 13 (E13; n = 49). Chi test values and statistical analyses are shown (**p ≤ 0.001). p value calculated by the Chi Test which determines p value based on degrees of freedom.

**Figure 2:**
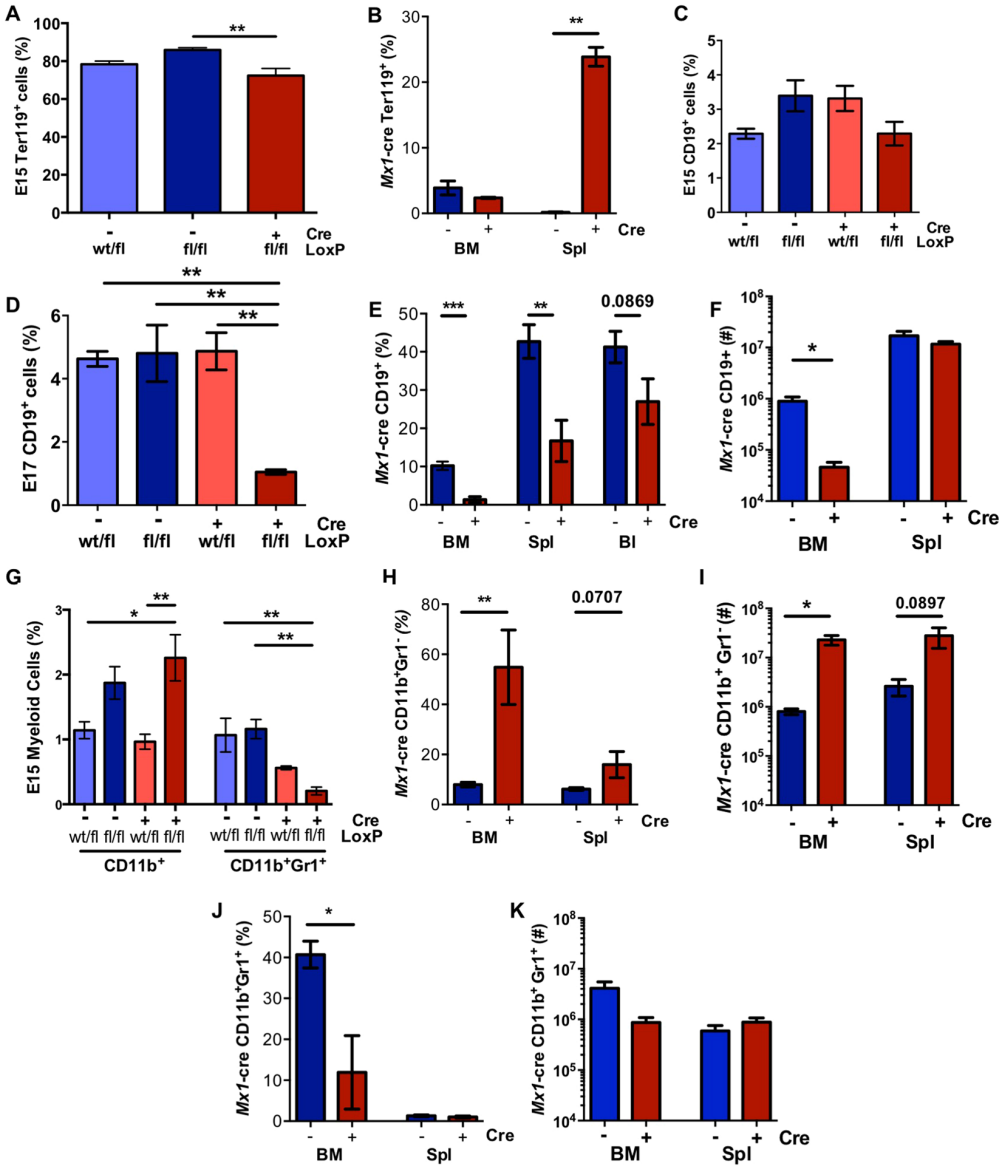
*Mx1/Vav*-cre^+^*Raptor*^*fl/fl*^ mice exhibit a disruption in haemopoiesis *in vivo*. Graphs showing the percentage of haemopoietic lineage calculated from flow cytometry data gated as indicated in Supplemental Fig. 1. Average percentage of Ter119^+^ erythroid population in *Vav*-cre^−^*Raptor*^*wt/fl*^*, Vav-Raptor* control, and *Vav-Raptor* KO E15 FLs (n = 7) **(A**) and BM and spleen of *Mx1*-*Raptor* control and *Mx1-Raptor* cKO mice (n = 3) **(B)**; Average percentage of CD19^+^ B cells in E15 (n = 6) **(C)** and E17 (n = 4) **(D)** FLs and BM, spleen and blood of *Mx1*-*Raptor* control and *Mx1-Raptor* cKO mice (n = 5) **(E)**; Average number of CD19^+^ B cells in BM and spleen of *Mx1*-*Raptor* control and *Mx1-Raptor* cKO mice (n = 5) (**F**); Average percentage of CD11b^+^Gr1^−^ (immature myeloid) and CD11b^+^Gr-1^+^ mature myeloid populations in *Vav-*control, and *Vav-*KO E15 FLs (n = 6) **(G)**, and CD11b^+^Gr1^−^ (immature myeloid) **(H)** and CD11b^+^Gr-1^+^ mature myeloid populations **(J)** in *Mx1-Raptor* control and *Mx1*-*Raptor* cKO BM and spleens (n = 5). Average number of CD11b^+^Gr1^−^ **(I)** and CD11b^+^Gr-1^+^ myeloid populations **(K)** in *Mx1-Raptor* control and *Mx1*-*Raptor* cKO BM and spleens (n = 5). All *Mx1*-cre mice were given 4 doses of poly(I:C) and assessed 5 wk post inoculation. Light blue bars - cre^−^*Raptor*^*wt/fl*^; blue *- cre*^*−*^*Raptor*^*fl/fl*^*;* red bars - cre^+^*Raptor*^*wt/fl*^*;* maroon bars - cre^+^*Raptor*^*fl/fl*^ as indicated. Data are expressed as mean ± SEM, one way ANOVA (p * ≤ 0.05; ** ≤ 0.001; *** ≤ 0.0001; **** ≤ 0.00001).

Analysis for the presence of selected haemopoietic lineages in these mouse models revealed a small but significant decrease in the percentage of Ter119^+^ erythroid populations in *Vav*-*Raptor* KO FL at E15 compared to *Vav*-*Raptor* control (Fig. 2A & Suppl Fig. 1A). Additionally, there was a trend in decrease in the percentage of Ter119^+^ erythroid populations in the BM, coupled with a significant increase in percentage of Ter119^+^ population in the spleen likely due to increased extramedullary haemopoiesis in *Mx1*-*Raptor* cKO mice compared to *Mx1-Raptor* control (Fig. 2B & Suppl Fig. 1B). Flow cytometric analysis of B lineage cells revealed a significant downregulation in the percentage of CD19^+^ B lineage cells in E17 *Vav-*KO FL and *Mx1*-cKO BM, spleen and blood (Fig. 2C–E; Suppl. Fig. 1), coupled with a significant decrease in the number of CD19^+^ B lineage cells in the *Mx1*-cKO BM (Fig. 2F). These data suggest a vital role for mTORC1 in B cell development. Both the KO models showed a concomitant increase in the percentage of CD11b^+^Gr1^−^ immature myeloid cells, together with a significant decrease in the percentage of CD11b^+^Gr1^+^ mature myeloid cells (Fig. 2G,H,J; Suppl. Fig. 1). These data were coupled with a significant increase in the number of CD11b^+^Gr1^−^ myeloid cells in the *Mx1*-cKO BM (Fig. 2I), while the mature myeloid cell numbers were unaltered in the *Mx1*-cKO BM and spleen (Fig. 2K). These data suggest that myeloid lineage maturation is deregulated in the absence of *Raptor* expression. As it is well established that mice can survive in the absence of white blood cell populations^22^, the absence of adult *Vav*-*Raptor* KO mice is likely due to the critical role of mTORC1 in erythropoiesis which results in the decrease in RBC populations and the pallor of the mice.

### mTORC1 plays a critical role in B and RBC lineage commitment *in vivo*

**Figure 3:**
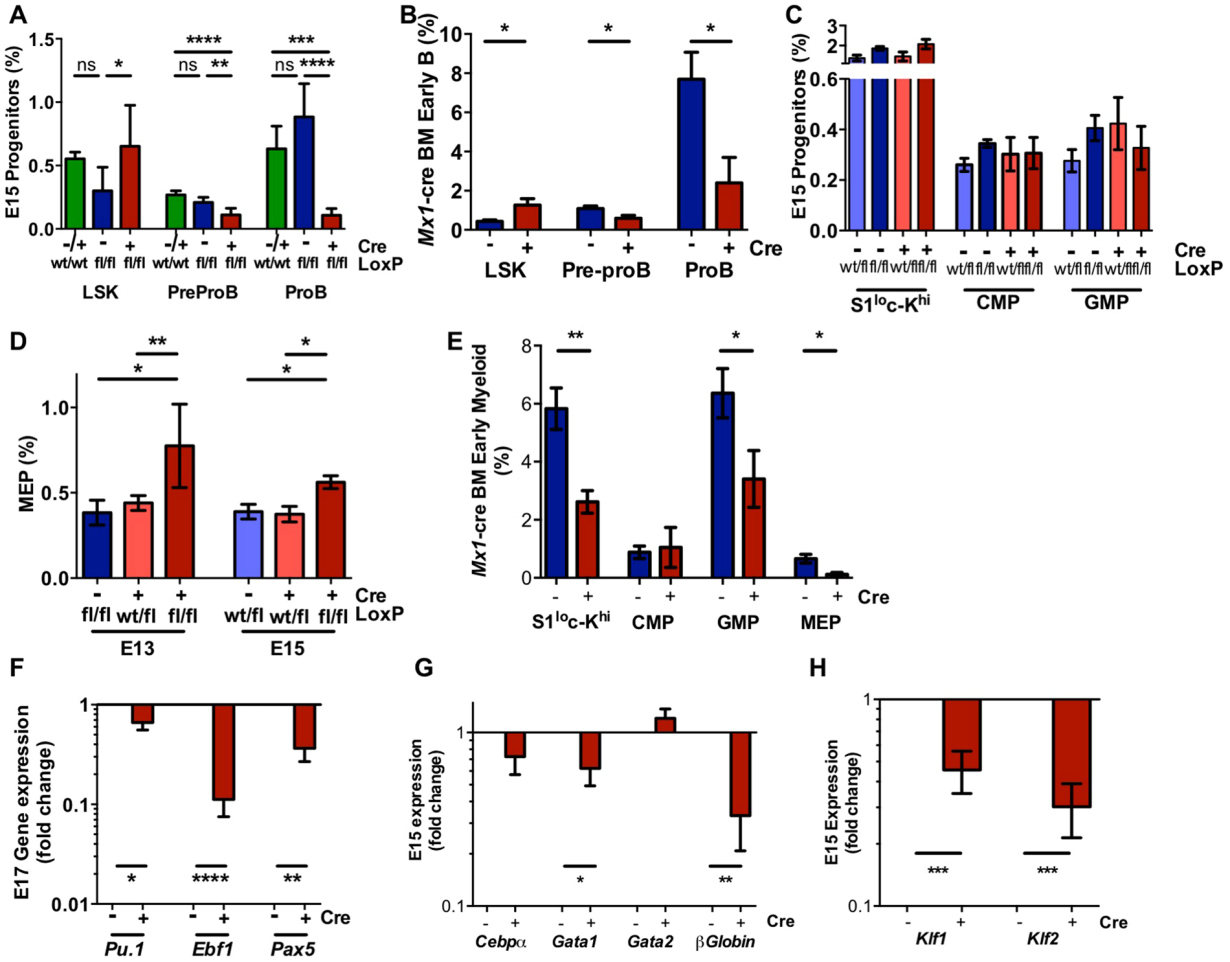
*Mx1* and *Vav-cre*^+^*Raptor*^*fl/fl*^ mice exhibit a block in B and RBC lineage commitment at the LSK and MEP stage *in vivo*. Surface expression of Lin^−^Sca-1^+^CD117^+^ (LSK), pre-proB (CD117^+^B220^+^CD19^−^) and proB (CD117^+^CD19^+^B220^+^) populations in E15 *Vav*-*Raptor* KO FL compared to *Vav*-cre^−^*Raptor*^*wt/fl*^ and *Vav*-cre^−/+^*Raptor*^*wt/wt*^ FL controls (n = 6) **(A)** and in BM of *Mx1*-*Raptor* control (blue) compared to *Mx1*-*Raptor* cKO (red) mice (n = 6) **(B)**; (**C**) Graph showing surface expression of S1^lo^c-Kit^hi^ (Sca-1^lo^CD117^hi^), CMP, GMP populations in E15 FL in *Vav-*cre^−^*Raptor*^*wt/fl*^ (light blue) and *Vav*-*Raptor* control (blue) and *Vav-*cre^+^*Raptor*^*wt/fl*^ (light red) and *Vav-Raptor* KO (dark red) (n = 6); (**D**) Surface expression of megakaryocyte erythrocyte progenitor (MEP) in E13 and E15 FL in *Vav-*KO mice compared to cre^+^*Raptor*^*wt/fl*^ and *Vav-*control (E13 n ≥ 3; E15 n ≥ 6); (**E**) BM of *Mx1*-control or *Mx1-*cKO mice showing surface expression of S1^lo^c-Kit^hi^, CMP, GMP and MEP populations (n ≥ 5); Gene expression of E17 FL demonstrating the fold change in *Pu.1*, early B cell factor 1 (*Ebf1*), and *Pax5* gene expression **(F)**, along with fold changes in gene expression of E15 FL *Cebpα*, *Gata1*, *Gata2*, *βGlobin* **(G)**, and Kruppel-like factor 1 and 2 (*Klf1* and *Klf2*) **(H)** in CD45^+^ sorted *Vav-Raptor* KO FL compared to *Vav-Raptor* control FL cells (n ≥ 4). All *Mx1*-cre mice were given 4 doses of poly(I:C) and assessed 5 wk post inoculation. Data are expressed as mean ± SEM, one way ANOVA (p * ≤ 0.05; ** ≤ 0.001; *** ≤ 0.0001; **** ≤ 0.00001).

To gain a deeper understanding of the stage at which *Raptor*-deficiency blocks lineage commitment/development we carried out flow cytometric analysis on the haemopoietic progenitor populations. Supporting the lack of CD19^+^ B cells, a significant reduction in the percentage of pre-proB and pro-B cells was noted in FL and BM isolated from *Vav*-*Raptor* KO and *Mx1-Raptor* cKO mice respectively, compared with control mice (Fig. 3A,B; Suppl. Fig. 2). This finding was coupled with a significant elevation in the Lin^−^Sca-1^+^CD117^hi^ (LSK) population, suggesting a block in B cell development prior to lineage commitment. Of note, no significant differences in LSK and progenitor B cell populations were observed between *VavCre*^*+/−*^*Raptor*^*wt/wt*^ and *VavCre*^*−*^*Raptor*^*fl/fl*^ mice, indicating that the phenotypic changes were due to the excision of *Raptor* (Fig. 3A). Analysis of the proportion of myeloid progenitors in E15 FL isolated from *Vav*-*Raptor* KO mice revealed no significant changes in the Sca-1^lo^CD117^hi^, CMP and GMP populations compared to the control mice (Fig. 3C; Suppl. Fig. 3A). An elevation in the percentage of MEPs in E13 and E15 FLs isolated from *Vav*-*Raptor* KO mice suggested a developmental arrest in erythropoiesis at the MEP stage in the absence of *Raptor* expression at the HSC stage *in vivo* (Fig. 3D). Interestingly, adult mice with an induced *Raptor-*deficiency exhibited a significant decrease in Sca-1^lo^CD117^hi^ population and MEP population suggesting a block in erythropoiesis prior to the MEP stage in *Mx1-Raptor* cKO BM (Fig. 3E). Additionally, a significant decrease in GMP population was noted, suggesting that differentiation of CMPs towards GMP and MEPs is deregulated in the BM in *Mx1-Raptor* cre cKO mice. The difference in progenitor populations between the FL and BM with *Raptor-*deficiency highlights the potential for mTORC1 to play similar but not identical roles in lineage maintenance at different stages of development and ontogeny.

In support of our phenotypic analyses showing a block in B cell and RBC development, analysis of key genes responsible for enabling differential lineage commitment revealed a significant downregulation in the expression of B cell specific TFs *Ebf1* and *Pax5* in FLs isolated from *Vav*-*Raptor* KO mice, while *Cebpα* levels were unaltered (Fig. 3F,G). Assessing erythroid lineage commitment factors, *Pu.1*, β-*Globin and Gata1* levels were significantly downregulated in *Vav*-*Raptor* KO mice with trends in an increase in *Gata2* levels. Additionally, there was a significant downregulation in the expression of *Klf1* and *Klf2*, TFs which play a role in embryonic erythropoiesis and development (Fig. 3H), indicating an aberration in erythropoiesis with *Raptor* deficiency in *Vav-*KO FLs.

### Inhibition of mTORC1 blocks RBC differentiation *in vitro*

**Figure 4:**
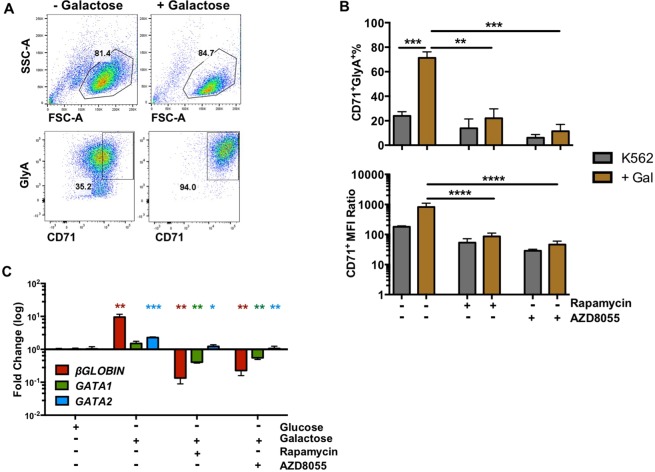
Role of mTORC1 in the differentiation of RBCs in a human cell model at the phenotype and gene transcription level *in vitro*. (**A**) Flow cytometry representative plot showing the levels of granularity (FSC-A vs. SSC-A) and in CD71^+^GlyA^+^ surface expression in K562 cells cultured in complete media or galactose-containing media. (**B**) Average percentage of CD71^+^GlyA^+^ cells and MFI ratio of CD71^+^ expression in K562 cells (n = 4) when glucose is replaced with galactose (golden bars) in K562 complete media (grey bars). (**C**) Gene expression of *β-GLOBIN, GATA1* and *GATA2* in K562 cells in complete media compared with cells with galactose treatment with or without mTOR inhibitors – rapamycin or AZD8055. Data (n = 4) are expressed as mean ± SEM, t-test (unpaired) (p * ≤ 0.05; ** ≤ 0.001; *** ≤ 0.0001; **** ≤ 0.00001).

To further assess the role of mTORC1 in RBC differentiation *in vitro*, we made use of the human BCR-ABL^+^ erythroleukaemia cell line K562, that differentiates towards a RBC-like lineage when exposed to stress^23–25^. Stress was induced by replacing glucose in complete media with galactose (Gal-media)^26^. A significant increase in the percentage of CD71^+^GlyA^+^ cells, an elevation of the erythroid marker CD71 surface expression and a reduction in cellular granularity was observed (Fig. 4A,B, Suppl. Fig. 4A) in K562 cells cultured in Gal-media, coupled with a significant increase in gene expression of β-*GLOBIN, GATA1* and *GATA2* (Fig. 4C). Treatment of K562 cells with either rapamycin (allosteric mTORC1 inhibitor) or AZD8055 (dual mTORC1/2 inhibitor) reduced erythroid differentiation *in vitro*, as indicated by a significant decrease in the generation of erythroid cells and a reduction in gene expression of erythroid markers, (Fig. 4B,C), indicating mTORC1 inhibition blocks RBC differentiation *in vitro*.

**Figure 5:**
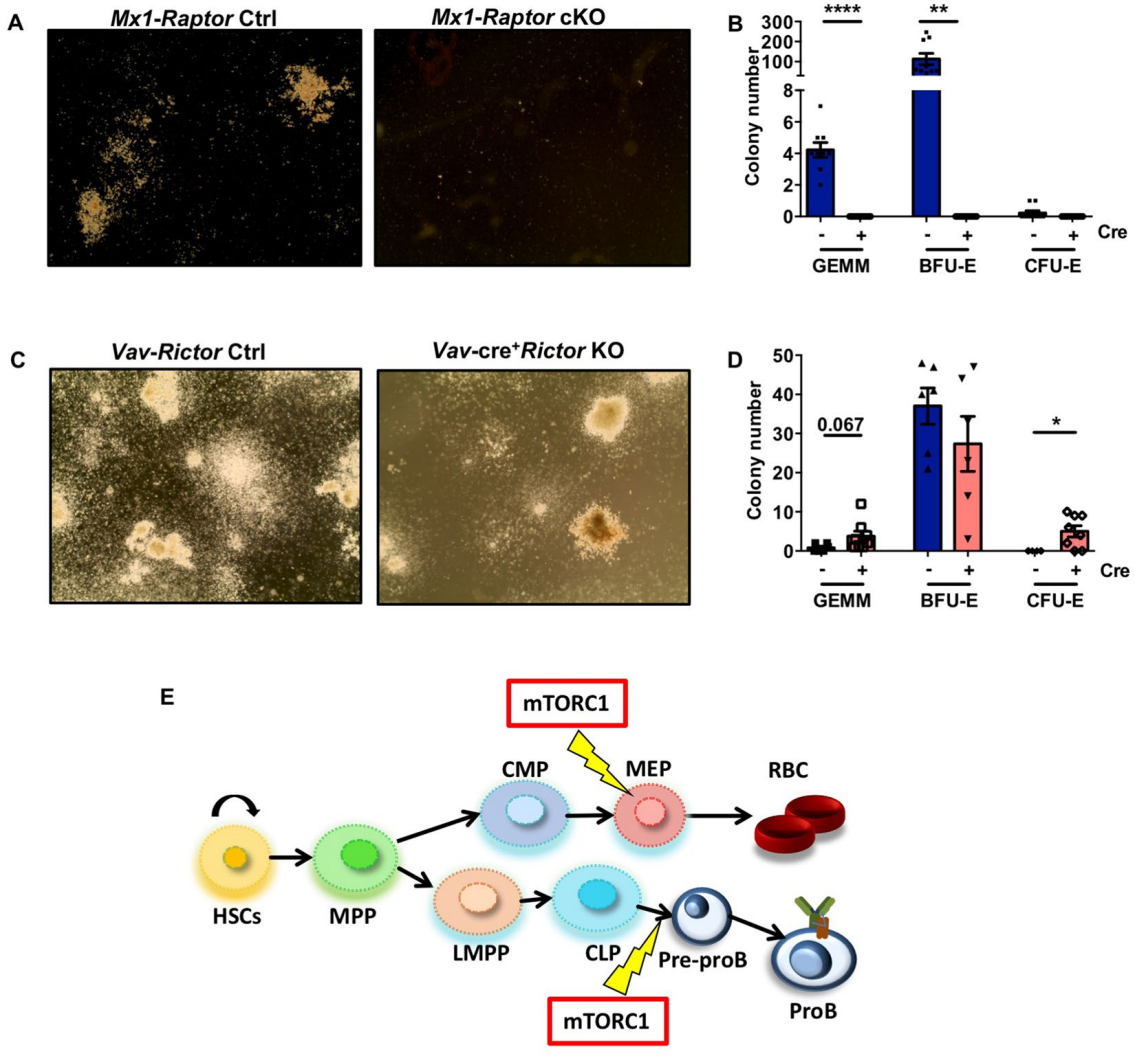
*Mx1*-*Raptor* cKO mice exhibit a functional block in RBC development. (**A**) Colony formation cell assays (CFCs) which optimise for the growth of erythroid cells were carried out on *Mx1*-*Raptor* control (control: left) and *Mx1*-*Raptor* cKO (cKO: right) BM enriched for haemopoietic stem/progenitor cells (HSPCs). All Mx1-cre mice were given 4 doses of poly(I:C) assessed 5 wk post last inoculation. (**B**) Colony counts of different erythroid colonies: colony-forming unit-erythroid (CFU-E), burst-forming unit-erythroid (BFU-E) and CFU of granulocyte-erythroid-macrophage-megakaryocyte (CFU-GEMM) in cre^+^*Raptor*^*fl/fl*^ KO models and in cre^−^ controls (n ≥ 6). (**C**) Colony formation cell assays (CFCs) which optimise for the growth of haematopoietic progenitor colonies were carried out on *Vav*-*Rictor* control (control: left) and *Vav*-*Rictor* KO (KO: right) BM enriched for HPCs. (**D**) Colony counts of different haematopoietic colonies: CFU-E, BFU-E and CFU-GEMM in cre^+^*Rictor*^*fl/fl*^ KO models and in cre^−^ controls (n ≥ 6). Data are expressed as mean ± SEM, unpaired t-test (p * ≤ 0.05; ** ≤ 0.001; *** ≤ 0.0001; **** ≤ 0.00001). (**E**) Schematic summary of the role of mTORC1 in B and erythrocyte development. Haemopoietic stem cells (HSCs), which have self-renewal capacity, give rise to multipotent progenitors (MPPs) which can develop towards common myeloid progenitors (CMPs), or lymphoid primed multipotent progenitors (LMPPs), which give rise to common lymphoid progenitors(CLPs) or certain myeloid cell lineages. CMPs further give rise to megakaryocyte-erythroid progenitors (MEPs) which develop into erythrocytes along with other lineages. CLPs give rise to lymphoid lineages including B cell lineage. Yellow lighting arrows represent stages of haemopoietic development where the role of mTORC1 is vital for further development.

To assess erythroid colony formation capacity of HPCs in the absence of mTORC1 activity, HPCs were isolated from BM of *Mx1*-*Raptor* control or cKO mice and CFC assays were performed. *Mx1*-*Raptor* cKO mice lacked CFC activity, as indicated by the lack of colony-forming unit-erythroid (CFU-E), blast-forming unit-erythroid (BFU-E) or granulocyte-erythroid-megakaryocyte-macrophage (GEMM) colonies in the absence of *Raptor* expression, compared to cre^−^ controls (Fig. 5A,B). Interestingly, myeloid progenitor CFC assays performed in *Vav*-cre^−^ (*Vav-Rictor* control) and cre^+^*Rictor*^*fl/fl*^ (*Vav-Rictor* KO) HPCs to assess the role of mTORC2 in early myeloid/erythroid colony formation demonstrated a significant increase in CFU-E colonies and a trend in increase in GEMM colonies in *Vav*-*Rictor* KO HPCs (Fig. 5C,D), suggesting a suppressive role of mTORC2 in erythropoiesis. Supporting these data, flow cytometric analysis of the CFCs generated showed a decrease in percentage of Ter119^+^ cells in *Mx1*-*Raptor* cKO HPCs, while an increase was noted in *Vav*-*Rictor* KO HPCs (Suppl. Fig. 5).

## Discussion

Our data support previously published work demonstrating a critical role of mTORC1 during B cell and erythroid development in two mouse models of *Raptor*-deletion *in vivo*, and further demonstrate the importance of mTORC1 function in *in vitro* RBC differentiation models: CFC assays and human cell line K562.

Examining the role of mTORC1 in normal erythropoiesis at the HSC stage, in both fetal and adult mice, revealed subtle differences at the two developmental stages. *Raptor-*deficient embryos were paler than *Raptor-*control mice, characteristic of mice with RBC deficiency as noted in EPO/EPO-R mice^27^. Knight *et al*., demonstrated high mTORC1 activity in proE cells (reticulocytes) characterised by elevated phosphorylation of the downstream target S6 thereby identifying a critical role for mTORC1 in RBC development. Additionally, they showed a link between iron availability and haemoglobin (Hb) synthesis in RBC development with mTORC1 signalling, as RBCs have decreased mTORC1 signalling during iron deficiency (ID) *in vitro* and *in vivo*^19^. We observed a significant decrease in Ter119^+^ erythrocytes, with an arrest at the MEP stage shown by a significant increase in the MEP population at E15, together with an increase in Sca-1^lo^ CD117^hi^ cells in the FL of *Vav*-*Raptor* KO. This is consistent with published data demonstrating *Vav-Raptor* KO FL exhibit microcytic anaemia with decreased Hb, exhibiting increased proE cells and decreased EryC populations. Conversely, constitutive activation of mTORC1 (*Vav-TSC1* KO) leads to macrocytic anaemia with larger reticulocytes and RBCs with elevated Hb levels. Despite increased Hb levels in RBCs, there was a decrease in RBC output in the BM (decreased EryB), leading to stress erythropoiesis in the spleen (elevated EryA population) with an overall decrease in Hb^19^. A steady decline in progenitor populations occurred over time, with no significant difference in MEP populations between the controls and *Vav*-*Raptor* KO FL at E17 (data not shown), diminishing any significant differences observed in FL at E13/E15. This is likely due to a transition in haemopoiesis from the FL to the BM, which begins before birth at E16.5^28^. The block in development of the erythroid lineage was supported by a significant downregulation in *Gata1, β-Globin, Klf1* and *Klf2* expression with *Raptor-*deficiency at the HSC stage, suggesting mTORC1-mediated signalling may aid in the regulation of GATA switching to enable erythropoiesis.

In contrast, induced *Raptor-*deficiency in adult mice (*Mx1-Raptor* cKO) is not lethal, and these mice displayed a significant decrease in Sca-1^lo^CD117^hi^ and MEP populations in the BM. This may be due to the lineage potential of progenitors differing between fetal and adult mice, as FL HSCs possess a higher proliferative and metabolic capacity (increased oxidative phosphorylation) than BM-derived HSCs which could affect Sca-1^lo^CD117^hi^ and MEP primitive populations^29^. Nevertheless, there is a trend in decrease in Ter119^+^ erythroid population in the BM of adult mice with *Raptor*-deficiency, as noted in the *Vav-*cre model, suggesting a disruption in erythropoiesis in the BM. Indeed, Guo *et al*., demonstrated that *Mx1-Raptor* cKO mice have reduced RBCs and Hb in the BM due to increased apoptosis of RBCs. Additionally, erythrocyte development was blocked at the proE stage^30^ confirming our results that mTORC1 blocks RBC development. *Mx1-Raptor* cKO mice exhibited splenomegaly, with the spleen showing a significant increase in Ter119^+^ population, which could suggest a possible compensatory mechanism leading to the promotion of erythropoiesis due to a lack of erythrocytes, suggesting extramedullary haemopoiesis in the spleen, as has been shown previously^31^. Alternatively, as the spleen is known to be a main site for erythrophagocytosis, it is possible that the function of these erythrocytes is compromised and are accumulated in the spleen for phagocytosis^30^. We observe splenomegaly and an increase in red pulp. As the red pulp is one of the major sites of erythrocyte destruction, it is possible that an accumulation of erythrocytes in the spleen indicates erythrocyte depletion^32^. The observed differences between the models may be due to the mechanism of *Raptor*-excision. While both models result in the deletion of *Raptor* in all haemopoietic lineages, the *Mx1-Raptor* cKO requires inoculation with poly(I:C), through induction of type 1 interferons, which may disrupt haemopoietic cell survival. Published studies indicate that the impact of poly(I:C) treatment on haemopoietic cells is transient, lasting for up to 48 hr^33^. Here, phenotypic analyses were carried out 5 wk after the last poly(I:C) inoculation, by which time the key poly(I:C) induced effect should be *Raptor*-excision. While this timeline may enable the outgrowth of undeleted alleles in *Mx1-Raptor* cKO mice, the optimisation of poly(I:C) induced *Raptor*-excision leading up to this study reduced the likelihood of this occurring in the timeframe of these experiments, and *Raptor* expression was consistently downregulated up to 8 wk post-poly(I:C) inoculation.

Using established mTOR inhibitors to block RBC differentiation in the K562 cell line provided support for the results presented from our mouse models, indicating that erythroid development is dependent on mTORC1 activity. Interestingly, a recent report shows that K562 cells express a distinct mTOR-containing complex, mTORC3 (mTOR associated with ETV7) that lacks other mTORC1/2 containing proteins, and is associated with rapamycin-resistance when upregulated in human cancer cells^34^. Our finding that K562 differentiation is inhibited by rapamycin treatment suggests that mTORC3 does not play a major role in this process. Supporting this, Ohyashiki *et al*. have previously shown that iron regulates mTORC1 signalling in RBCs as iron chelators greatly reduced mTORC1 signalling in K562 cells^35^. Overexpression of eIF4E and sustained mTORC1 expression in erythroid progenitor cell line I/11 also improves differentiation^36,37^. An additional role of mTORC1 in initiation of translation at early stages in RBC development has been reported, demonstrating the regulation of mitochondrial biogenesis from HSPCs to proerythroblasts by mTORC1^38^. This study shows a unique requirement for mTORC1 in RBC development. These data, together with our findings suggest that mTORC1, iron metabolism and erythropoiesis have an inter-dependent regulatory link.

Assessment of erythroid colony formation capacity revealed that *Raptor-*deficient BM lacked colony formation capacity. Consistent with this, *Mx1-Raptor* cKO cells have previously been shown to lack the ability to form CFU-E and BFU-E colonies^30^. Indeed, shRNA targeting S6K1 in murine BM cells significantly decreased CFU-E and BFU-E colony formation capacity demonstrating the importance of mTORC1-S6K axis is mediating erythropoiesis^39^. *ER*-*Raptor* cKO LSK cells also lack colony formation capacity which is rescued by the retroviral transduction of *Raptor* suggesting the importance of mTORC1 in haemopoiesis^40^. Our assessment of *Rictor-*deficient BM colony formation capacity revealed that *Vav-Rictor* KO BM formed a significantly higher number of erythroid colonies than their controls. This suggests a possible feedback system for erythroid regulation wherein mTORC1 drives erythroid formation, whereas mTORC2 suppresses erythropoiesis. Our study compared the *Mx1-Raptor* cKO and *Vav-Rictor* KO models. *Vav*-mediated KO models generate a constitutive KO, which can result in signalling pathway adaptation^41^. Indeed, crosstalk between ERK-MAPK and mTOR signalling pathways has recently been demonstrated, with ERK activity regulating mTORC1 activation to limit the promotion of HSC cycling in favour of quiescence. HSCs derived from MEK1-cKO mice exhibit exhaustion due to increased mTORC1-mediated ROS production resulting in increased mitochondrial damage^42^. While each model has a different timing for gene deletion, the cells placed in CFC assays were removed from *Mx1-Raptor* mice 5 wk after inoculation with poly(I:C) *in vivo*, thus inducing optimal *Raptor* excision.

Of note, publications also suggest that mTORC1 negatively regulates erythropoiesis, via heme-regulated eIF2α kinase (HRI) and activation transcription factor 4 (ATF4), in a mouse model of ID. HRI is activated in response to heme deficiency, thereby stimulating ATF4 to regulate Hb levels via the translation of stress response genes. In mutant mice that lack phosphorylated eIF2α (eIF2αP) in the RBC lineage (*eAA*)^43^, mTORC1 activity is elevated in erythroid precursors. This study demonstrated that eIF2αP and ATF4 of HRI-integrated stress response are required in ID to repress mTORC1 signalling and mitigate ineffective erythropoiesis. As such, pharmacological inhibition of mTORC1 significantly improved RBC production and differentiation, and increased Hb levels in the blood^43^. Furthermore, constitutive activation of mTORC1 in haemopoietic lineages exhibited macrocytic hyperchromic anaemia with splenomegaly and ineffective erythropoiesis^19^, similar to that seen in HRI^−/−^ mice. However, although ATF4 is required to inhibit mTORC1 signalling, ATF4^−/−^ mice develop microcytic hypochromic anaemia, unlike HRI^−/−^ mice^43^. These studies suggest that mTORC1 activity plays an inhibitory role in erythropoiesis during ID anaemia. Furthermore, *Foxo3*^−/−^ mice (model with similar genotypic profile to β-thalassemia), exhibit increased phosphorylation of mTOR targets including AKT, S6, 4EBP1, suggesting aberrant mTOR signalling in diseased conditions. Treatment of *Foxo3*^−/−^ mice with rapamycin increased RBC counts and Hb levels, with increased cell cycling in immature erythrocytes compared to untreated mice^39^. Therefore, in contrast to normal erythropoiesis, where mTORC1 plays a fundamental role in RBC generation, diseased conditions alter mTORC1 signalling to disrupt erythropoiesis, which is overcome by mTORC1 inhibitors.

The early block in B cell lineage commitment and development in the FL and BM of both *Raptor*-deficient models, mirrored that described previously in *Mx1-Raptor* cKO mice^31^. The observed increase in LSK populations, which normally give rise to pre-proB cells, in both BM and E15 FL suggests a block at the LSK stage during B cell development with *Raptor-*deficiency at the fetal and in adult stages of development. These findings were supported by the observed downregulation in *Ebf1* and *Pax5*, TFs essential for B cell lineage commitment. A similar trend has been observed in the BM of *mTOR*-cKO mice, and the spleen of *Mx1-Raptor* cKO mice, with the majority of LSK cells in S phase suggesting increased cell cycling due to perturbations in mTORC1 signalling^29,30^. Iwata *et al*., demonstrate a block in B cell development at the pre-B cell stage within *Mb1-Raptor* cKO adult mice, which is not rescued by the introduction of an anti-apoptotic *Bcl*_*XL*_ transgene suggesting that this block, caused by the lack of mTORC1, is independent of BCL_XL_^44^. Our model, which excises *Raptor* at the HSC stage, demonstrates a requirement for mTORC1 signalling for B cell lineage commitment. In *ER*-*Raptor* cKO BM there is an increase in LSK cells together with a decrease in phosphorylation of 4EBP1 and S6 and decrease in mature B cells^40^ indicating that the role of mTORC1 in B lymphopoiesis is not model specific. Indeed, Zeng *et al*. highlight the importance of mTORC1 specifically in B cell development and proliferation, but not survival, in an IL7R-mTORC1-Myc dependent and STAT5-independent manner, as the loss of mTORC1 leads to a block at the proB cell stage in *CD2-mTOR* and *CD2-Raptor* cKO models^45^. Furthermore, Keating *et al*. demonstrated a critical role for mTORC1 in B cell class switching and anti-viral responses, with *Rosa26-Raptor* KO mice having reduced germinal centre formation in response to influenza infection, a finding that was replicated with rapamycin treatment. Interestingly, rapamycin treatment prevented B cell class-switching, yielding antibodies that mediated heterosubtypic protection, by targeting more conserved regions of hemagglutinin, a surface protein expressed on viruses^46^. H&E staining of spleens isolated from *Mx1-Raptor* cKO mice lack a defined architecture and displayed a reduction in marginal zones and germinal centres (data not shown).

In agreement with previously published data, similar roles of mTORC1 in myeloid lineage development were observed in both fetal and adult mice with a significant increase in CD11b^+^Gr1^−^ populations and a concomitant decrease in CD11b^+^Gr1^+^ populations in both *Vav-Raptor* KO FL and *Mx1*-*Raptor* cKO BM^30,31^. This result has been recapitulated in *ER-*cre model suggesting that the role of mTORC1 in myeloid development is not model specific^40^. We observed a decrease in GMP population leading to a decrease in mature myeloid population with *Raptor-*deficiency in *Mx1*-*Raptor* cKO mice. Indeed, a decrease in CMPs has previously been reported in *Mx1-Raptor* cKO mice^30^ supporting the decrease in mature myeloid lineage with *Raptor-*deficiency. However, the increase in CD11b^+^ population in both models also suggests that even though there is a block in early myeloid populations, it does not disrupt the ability of the progenitors to develop into immature myeloid cells. *ER-Raptor* cKO mice displayed an increase in GMP and MEP populations with a block in CD11b^+^Gr1^+^ mature myeloid populations with no changes in pS6, alluding to the phenotypic differences between KO models. The lack of change in pS6 expression in myeloid cells (with a significant decrease in pS6 in B cells) suggests a differential regulation of mTORC1 activity in different haemopoietic lineages^40^. Nonetheless, mTORC1 plays a role in the maturation of myeloid cells in both adult and fetal models. While previous studies support a cell autonomous effect of *Raptor*-deletion in the development of haemopoietic lineages^30,31^, mixed BM chimeras should be performed to confirm our results, as myeloid lineage expansion has the potential to inhibit erythroid and lymphoid lineages^47,48^. In addition, deletion of *Raptor* in HSCs has been shown to affect osteoclast generation, which in turn would impact the BM microenvironment and subsequent haemopoiesis^49^.

Taken together, we performed analyses in two different mTORC1 KO mouse models at different stages of development *in vivo* and two distinct RBC differentiation assays *in vitro* to tease out the role of mTORC1 in haemopoietic lineage development. We confirm the fundamental role of mTORC1 in haemopoiesis, during RBC and B cell lineage commitment both in fetal and adult mice (Fig. 5E).

## Materials and Methods

### Mice

Mice expressing floxed *Raptor* (*Raptor*^*fl/fl*^) or *Rictor* (*Rictor*^*fl/fl*^) constructs were obtained from Prof. Michael N. Hall (University of Basel, Switzerland)^50^. Prof. Tessa L. Holyoake (University of Glasgow, UK) provided transgenic mice expressing *Mx1*-cre and *Vav*-cre, which were bred and maintained at the Beatson Research Unit (Glasgow, UK). All experimental protocols were approved by the local AWERB committee and national home office, and all methods were carried out in accordance with standard animal housing conditions under local and home office regulations. *Mx1*-cre^−/+^ or *Vav*-cre^−/+^ transgenic mice were crossed with *Raptor*^*fl/fl*^ or *Rictor*^*fl/fl*^ to obtain the desired KO models. The *Mx1* promoter was activated in *Mx1*-cre^−/+^-*Raptor*^fl/fl^ or -*Rictor*^*fl/fl*^ mice by inoculating the mice with 4 doses of 10 mg/kg polyionosinic:polycytidylic acid (poly(I:C); TLR3 agonist) once every 2 days to induce a conditional KO (cKO). The mice were assessed 5 wk after the last inoculation. No significant differences were observed in organ cellularity, and B/myeloid lineage percentage and number when B6.SJL wild type mice and *Mx1*-Cre^−^*Raptor*^*fl/fl*^ mice were compared after treatment with poly(I:C); Supplemental Fig. 6). The *Vav* promoter is active at E13 in HSCs, thereby inducing a haemopoietic-restricted KO. Haemopoietic lineage cells in a single cell suspension were isolated from BM, spleen and blood of transgenic mice, and filtered through a 70 µm nylon mesh (Fisher Scientific, Leicestershire, UK). The cells collected from the BM and spleen were enriched for haemopoietic lineage cells by density centrifugation using Lympholyte-Mammal (Cedarlane, Canada), centrifuging the cell suspension for 20 min at 625 *g* at RT. Thereafter the cells were washed in PBS, centrifuged at 500 *g* for 10 min at RT and counted.

### Flow cytometry

Samples derived from the organs were prepared for flow cytometry as described previously^51^. All antibodies were purchased from BD Biosciences (Oxford, UK), except Gr-1 (Clone RB6-8C5; eBiosciences) and Ter119 (Clone TER-119; BioLegend). K562 cells with/without treatment were harvested and 2 × 10^5^ cells were stained with GlyA (Clone GAR2) and CD71 (Clone AC102) from Miltenyi Biotec. All samples were suspended in 100 µl PBS and acquired using the FACSDiva software package (BD Biosciences) on the FACSCantoII flow cytometer. The resultant data were analysed using FlowJo software (Tree Star Inc., OR).

### qRT-PCR

RNA was extracted from fresh cells by following the RNAeasy Qiagen Kit protocol. cDNA was then made from the RNA using standard protocols provided by Invitrogen. Alternatively, CD45^+^ cells were sorted into the PCR tubes (300 cells/tube) containing the one-step PCR master mix of 5 µl (Cells direct 2x reaction (2.8 µl), 0.2x Primer mix (1.4 µl), RNAase out (0.056 µl), Superscript III RT/Platinum Taq Mix (0.112 µl), TE Buffer (0.672 µl) (part of SuperScript™ III Platinum™ One-Step qRT-PCR Kit, Invitrogen)). Samples were run on a PCR with the following conditions: 50 °C for 15 min, 95 °C for 2 min followed by 20 cycles of 95 °C for 15 sec and 65 °C for 4 min. The samples were then held at 4 °C. After the completion of the PCR, the samples were diluted 10x with TE buffer (45 µl). qPCR was carried out by using 300 nM of forward and reverse primer for each gene. All reactions were performed in technical triplicates and at least three biological replicates using the 7900HT Fast Real-Time PCR system (Applied Biosystems, Warrington, UK), programmed to complete 40 cycles as follows: 50 °C for 20 min, 95 °C for 10 min, followed by 40 cycles of 95 °C for 15 sec, and 60 °C for 1 min. The primers used are listed in Supplementary Table 1. The q/RT-PCR follows the MIQE guidelines^52^.

### Erythroid differentiation of K562 cells

For normal cell growth, K562 cells were cultured in RPMI-1640 supplemented with 10% FBS, 50 U/ml penicillin, 50 µg/ml streptomycin and 2 mM L-Glutamine (complete media). To induce erythroid differentiation, K562 cells were cultured in RPMI-1640 (no glucose) supplemented with 10% FBS, 50 U/ml penicillin, 50 µg/ml streptomycin, 2 mM L-Glutamine and 11 mM Galactose (Gal-media). For erythroid differentiation, K562 cells (2 × 10^5^) were cultured in either complete media or Gal-media for up to 7 days and then analysed by flow cytometry or qPCR. For drug treatments, cells were treated with 10 nM rapamycin (allosteric mTORC1 inhibitor) or 100 nM AZD8055 (competitive dual mTORC1/2 inhibitor) or vehicle control (DMSO) as indicated.

### Colony forming cell (CFC) assay

Bones from transgenic mice were crushed and enriched for haemopoietic lineages as described above. Haemopoietic progenitor cells (HPCs) were isolated using the MACS mouse CD117 MicroBeads according to the manufacturer’s protocol (Miltenyi Biotec, Surrey, UK). Up to 2 × 10^4^ cells were then plated in MethoCult^TM^ M3334 erythroid colony promoting methylcellulose or MethoCult^TM^ GF M3434, in 6 cm dishes as per the manufacturer’s protocol (Stemcell Technologies, Grenoble, France). The dishes were monitored and colonies were counted.

### Statistics

The Chi Test was used to assess whether there were statistical differences between the different genotypes generated from crossing *Vav*-cre^−/+^*Raptor*^*fl/fl*^ mice. Chi Test calculates deviations by chance if the expected ratios are known. Medelian ratios were the expected ratios, which were compared to the genotypes observed at weaning stage of mice. The statistical significance, if any, is calculated using the Chi Test taking into account the degrees of freedom^53^. Statistical analyses were carried out between the data sets using GraphPad Prism 6 Software (San Diego, California, USA). Statistical analyses carried out were unpaired t tests, or for data with multiple comparisons one way ANOVA, where p ≤ 0.05 is considered significant. * ≤ 0.05; ** ≤ 0.001; *** ≤ 0.0001; **** ≤ 0.00001.

## Supplementary information

Supplemental Data

## Data Availability

No datasets were generated or analysed during the current study.
