# Supplementary material for: mTORC1 activity is essential for erythropoiesis and B cell lineage commitment
Source: Sci Rep. 2019 Nov 15;9:16917. doi: 10.1038/s41598-019-53141-1 (PMC6858379; doi:10.1038/s41598-019-53141-1)

**Supplemental Data File for:**

**mTORC1 activity is essential for erythropoiesis and B cell lineage commitment**

Natasha Malik^1^, Karen Dunn^1^, Jennifer Cassels^1^, Jodie Hay^1^, Christopher Estell^1^, Owen J. Sansom^1,2^, Alison M. Michie^1^

^1^Institute of Cancer Sciences, College of Medicine, Veterinary and Life Sciences,

University of Glasgow, Glasgow UK; ^2^Cancer Research UK Beatson Institute, Garscube Estate, Glasgow UK.

| Gene | Forward | Reverse | Species | Sequence Information |
| --- | --- | --- | --- | --- |
| *Cebpa* | aagaacagcaacgagtaccg | ttctgttgcgtctccacgtt | Mouse | NM_001287521.1 |
| *Ebf1* | tacagaaggtcattcctcgg | atcccatacagggcttcaac | Mouse | NM_001290709.1 |
| *Gata1* | atgattgtcagcaaacgggc | aggcattgcataccggatct | Mouse | NM_008089.2 |
| *Gata2* | gacgacaaccaccaccttat | ggtcagtggcctgttaacat | Mouse | NM_008090.5 |
| *Gusb* | taagacgctgatcacccaca | cagataacatccacgtacgg | Mouse | NM_010368.1 |
| *mHba-a1* | aacttcaagctcctgagcca | tgctcaagaggcaaggaat | Mouse | NM_008218.2 |
| *Klf1* | ctaagaggcaggcggcacat | ctgagcgagcgaacctcc | Mouse | NM_010635.3 |
| *Klf2* | ccaagagctcgcacctaaag | gtggcactgaaagggtctgt | Mouse | NM_008452.2 |
| *Pu.1* | cagcgatggagaaagccata | ctctgtgaagtggttctcag | Mouse | NM_011355.2 |
| *Rptor* | atggtagcaggcacactcttcatg | gctaaacattcagtccctaatc | Mouse | Ref. 50 |
| *Tbp* | gtacccttcaccaatgactc | cagccaagattcacggtaga | Mouse | NM_013684.3 |
| *HBB* | gcaaggtgaacgtggatgaa | agcactttcttgccatgagc | Human | NM_000518.4 |
| *GATA1* | tattcctctcccaagcttcg | catcttgtgatagaggccgca | Human | NM_002049.3 |
| *GATA2* | gacgacaaccaccaccttat | ggtcagtggcctgttaacat | Human | NM_001145661.2 |
| *RNF20* | ggtgtctcttcaacggaggaa | tagtgaggcatcatcagtggc | Human | NM_019592.7 |

Supplementary Table 1: List of primers used for PCR reactions.

The full sequence for each gene was obtained from PubMed website. Each primer was designed to have close to 10 C=G and 10 A=T bonds. The length between the forward and reverse primer is between 150-300 base pairs. *RNF20* was used as a reference gene for K562 cell line. *Gusb* and *Tbp* were used as reference genes for murine cells. The specificity of each primer sequence was checked by using BLASTn tool.


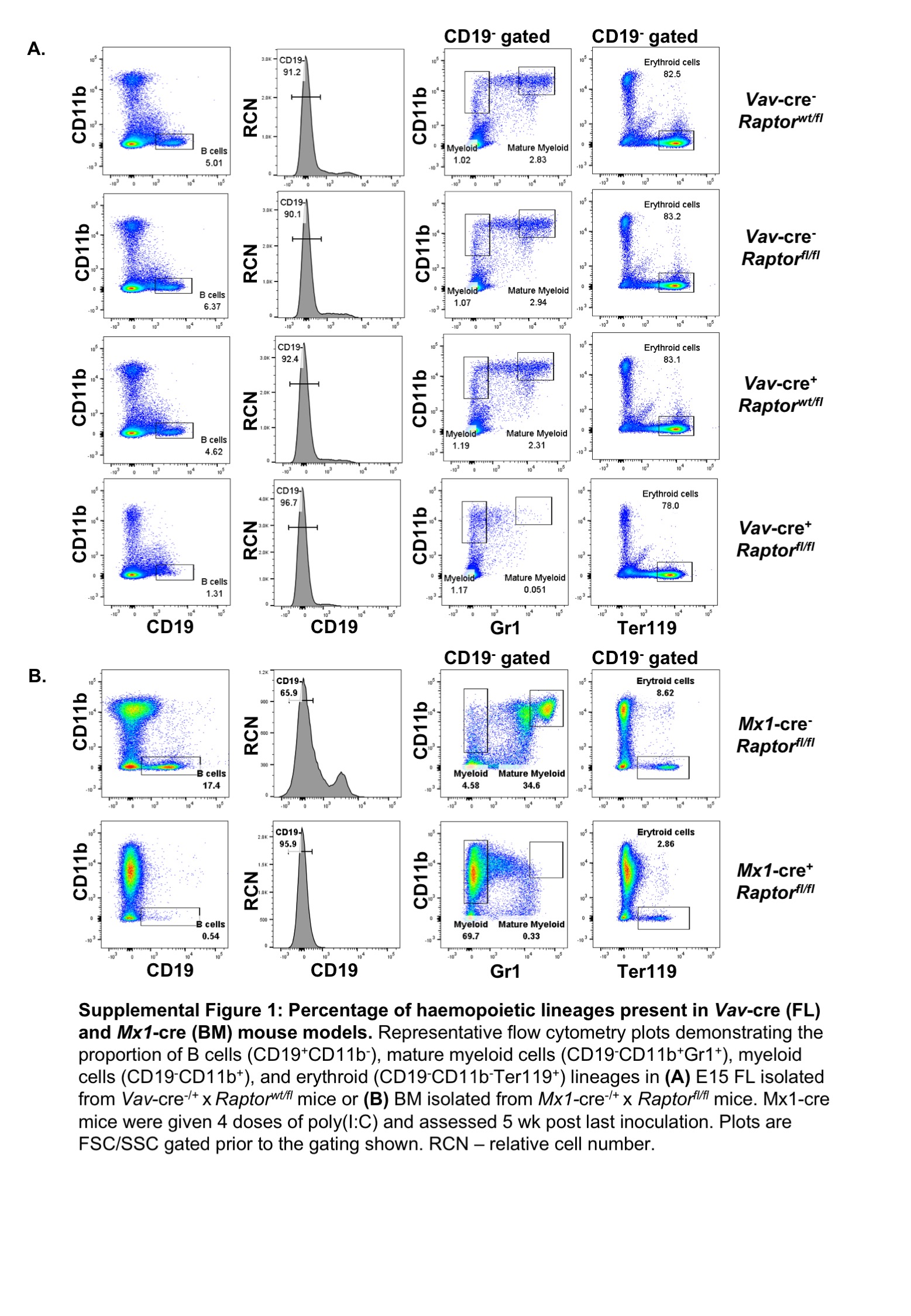


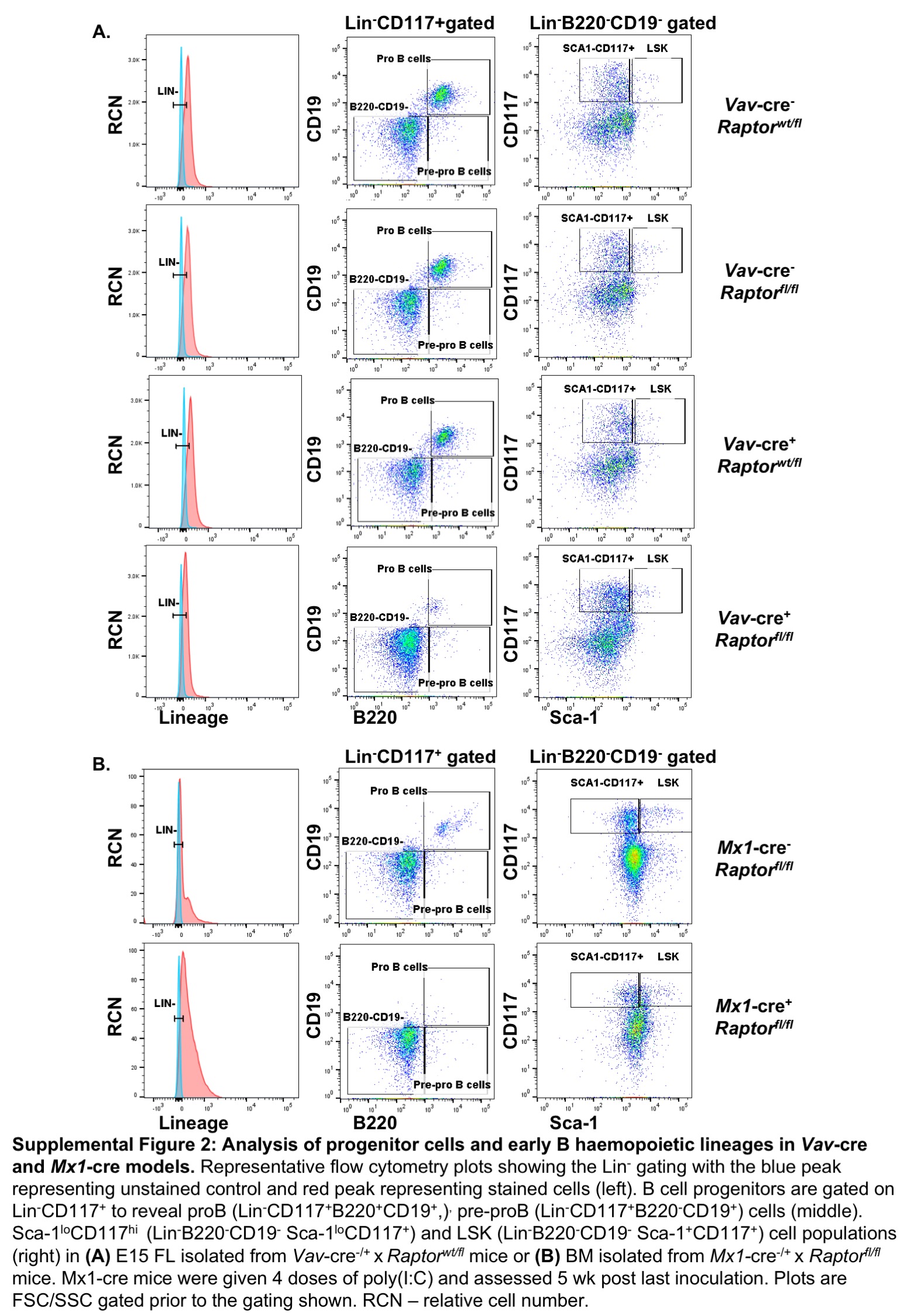


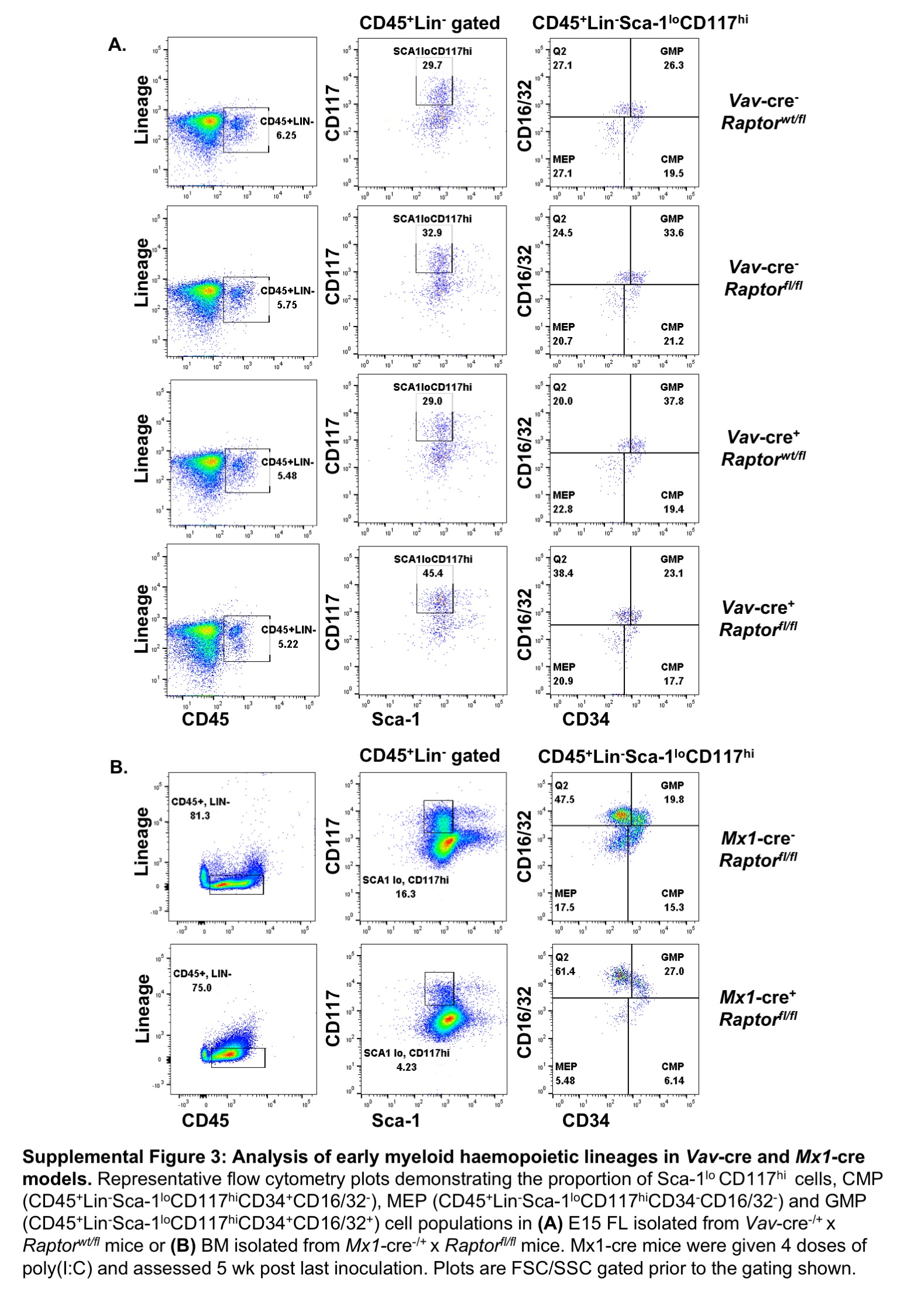


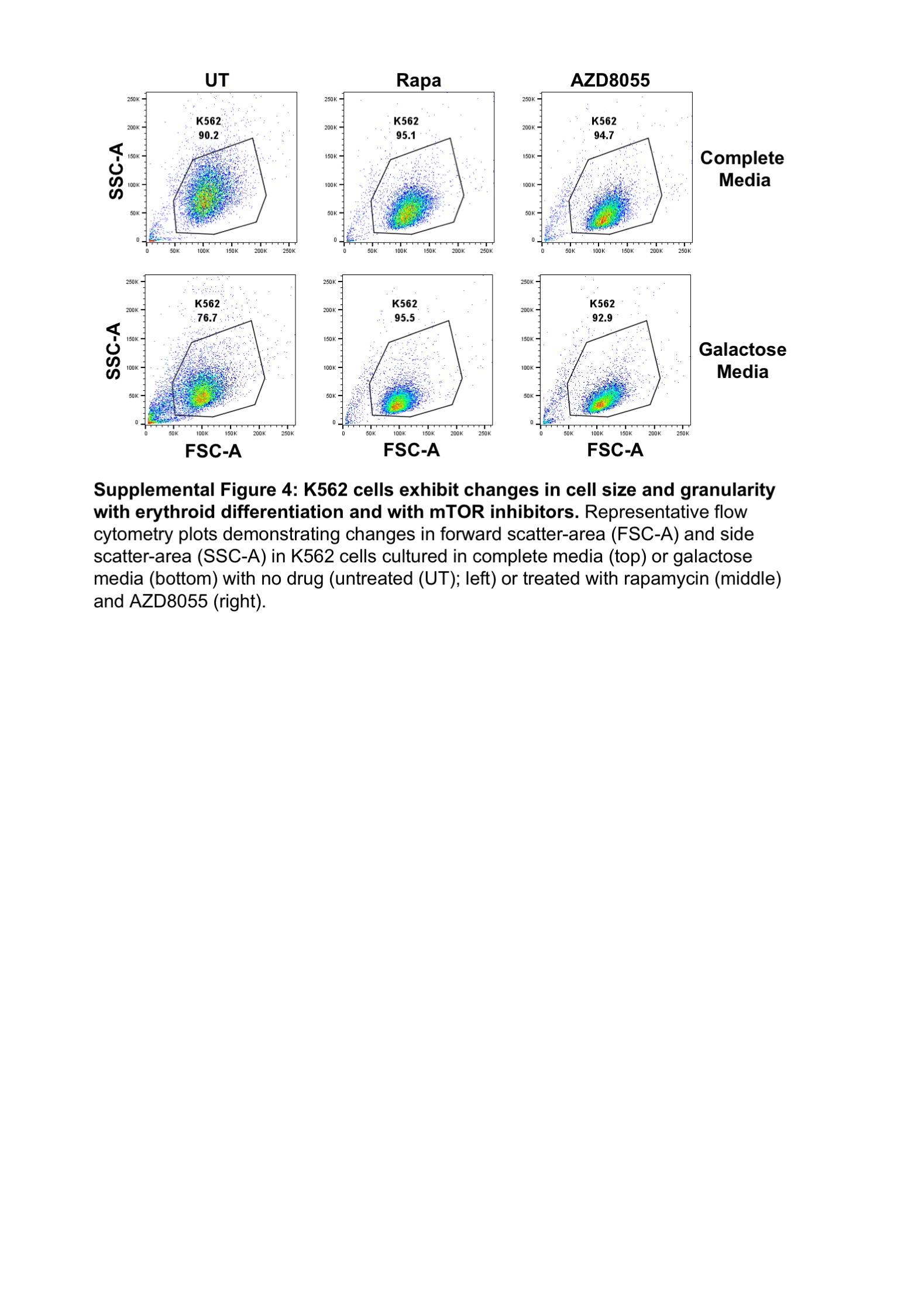


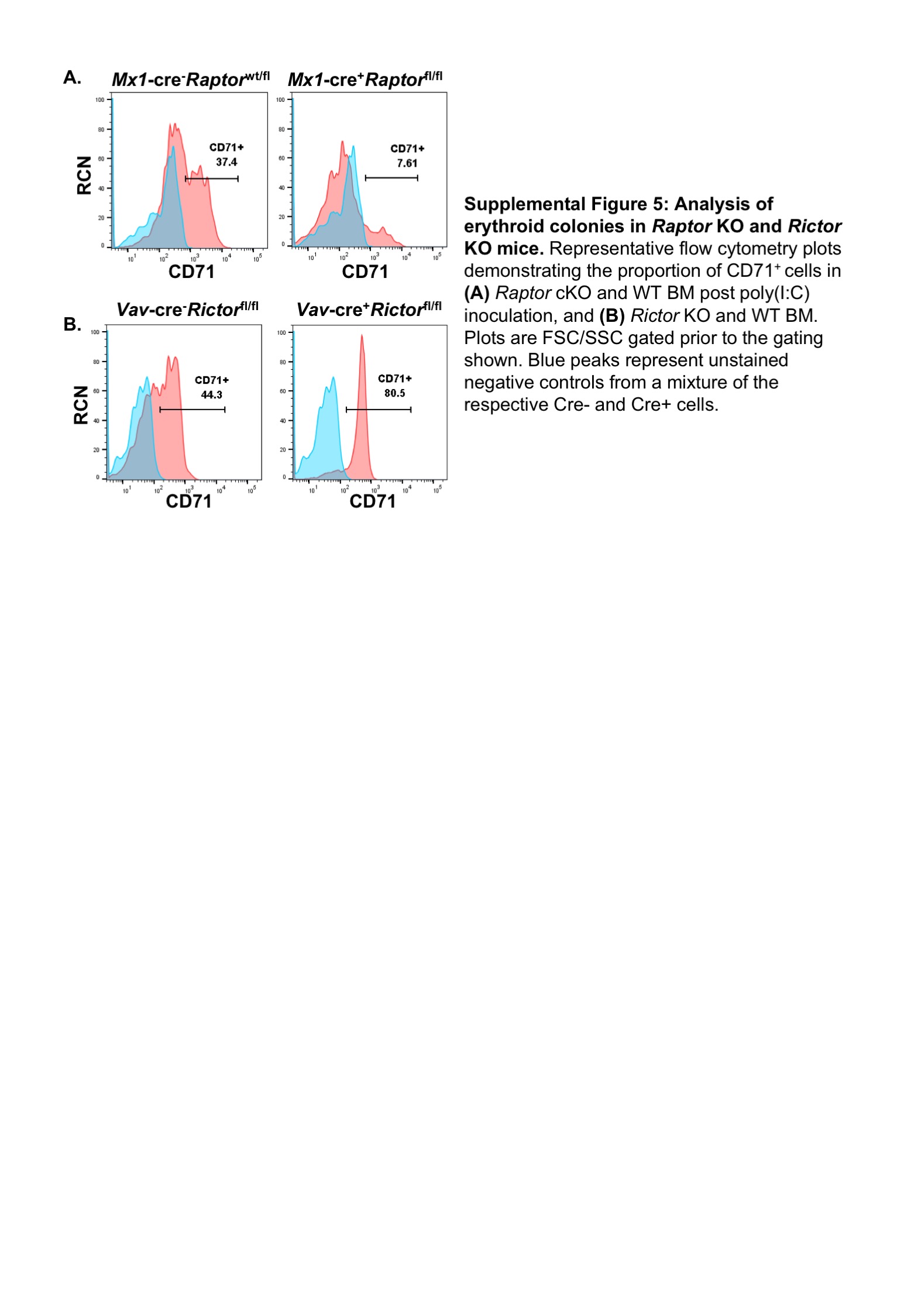


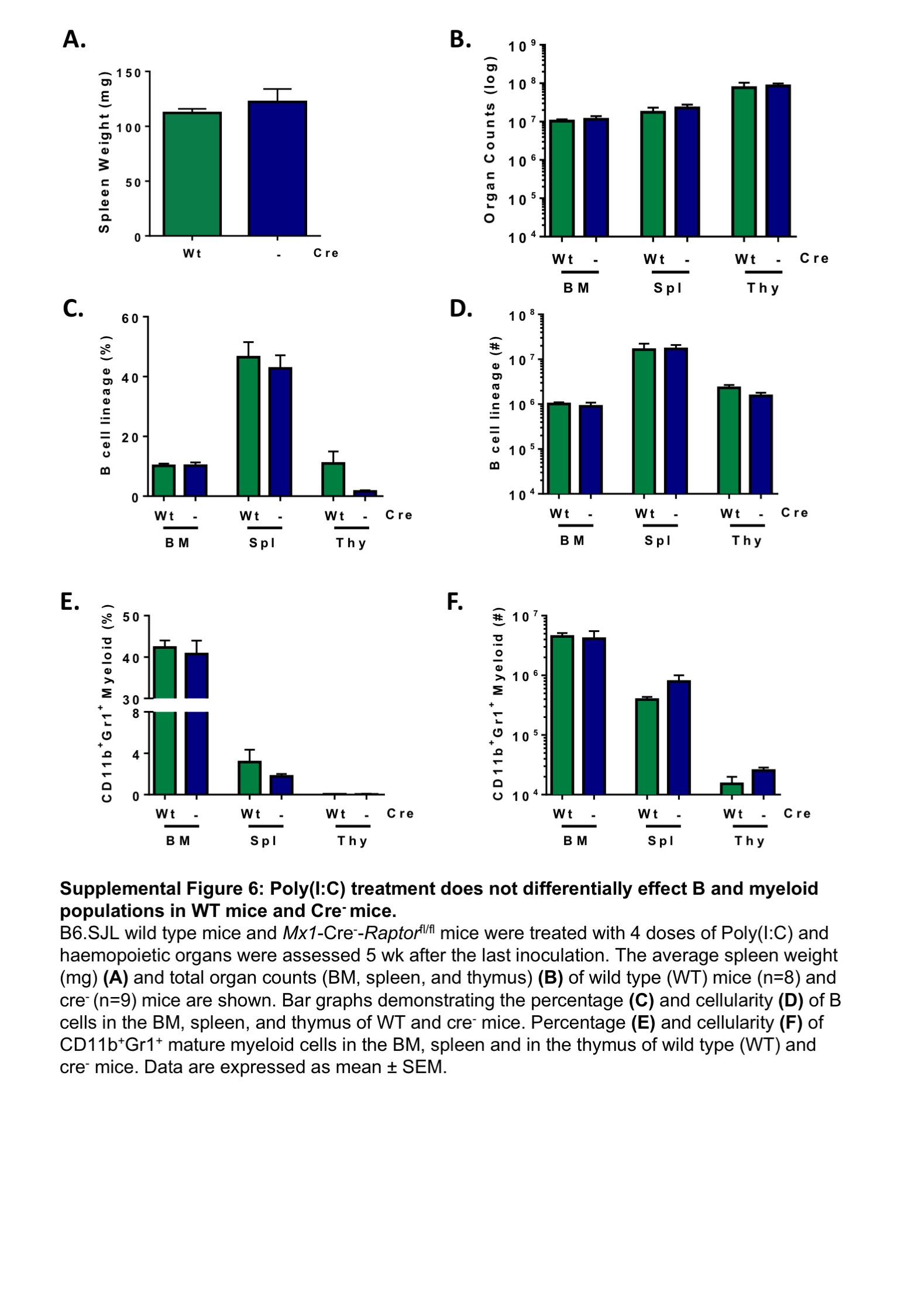

Supplement: Supplementary file 1 — Supplemental Data [file 41598_2019_53141_MOESM1_ESM.docx]
